# Supplementary figures and images for: Small RNA Sequencing Analysis of STZ-Injured Pancreas Reveals Novel MicroRNA and Transfer RNA-Derived RNA with Biomarker Potential for Diabetes Mellitus
Source: Int J Mol Sci. 2023 Jun 19;24(12):10323. doi: 10.3390/ijms241210323 (PMC10298999; doi:10.3390/ijms241210323)

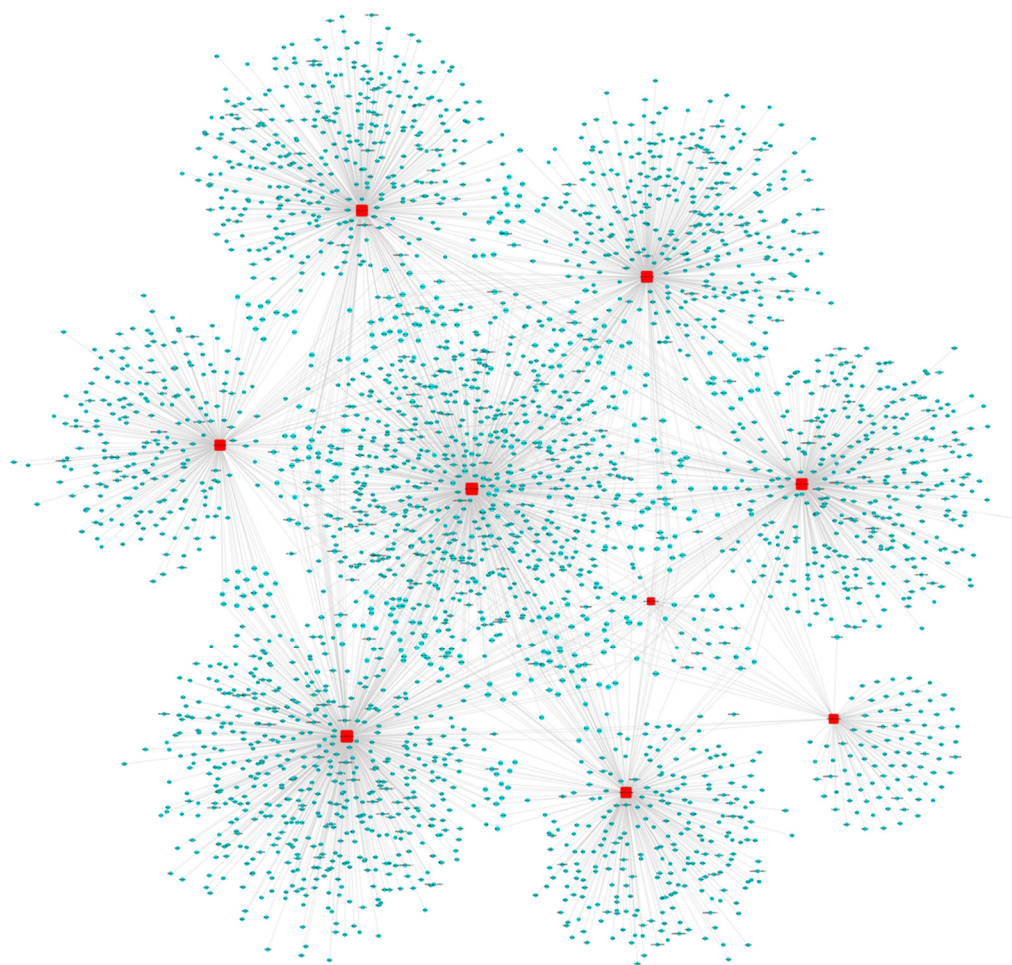

Figure S1\_up\_mirTarget

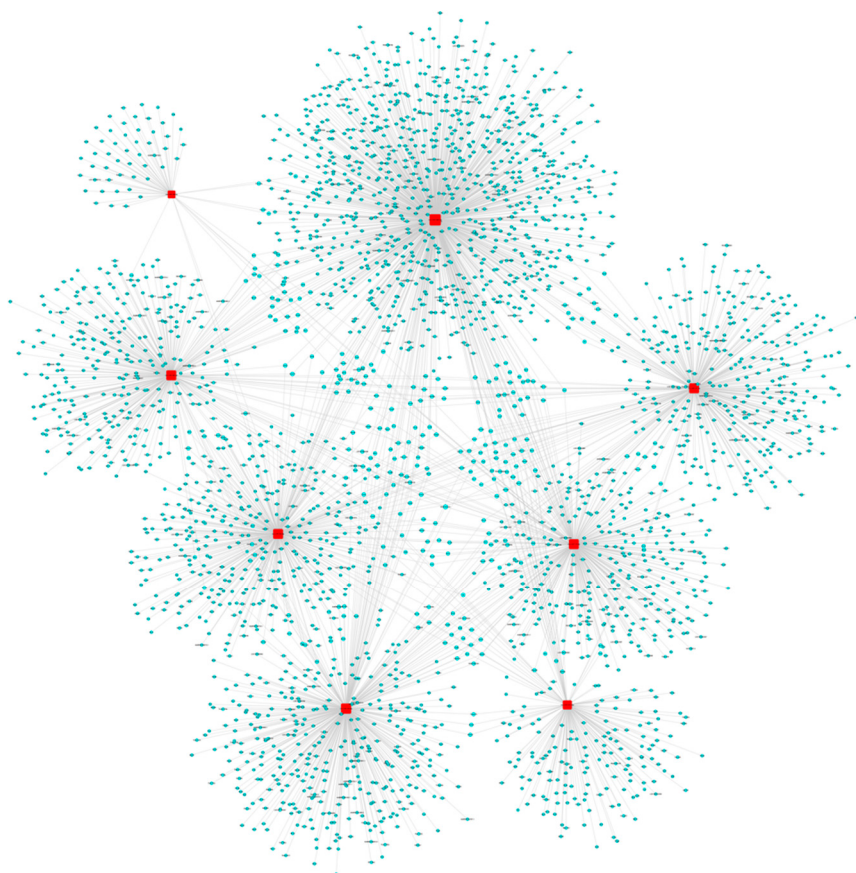

Figure S2\_down\_mirTarget

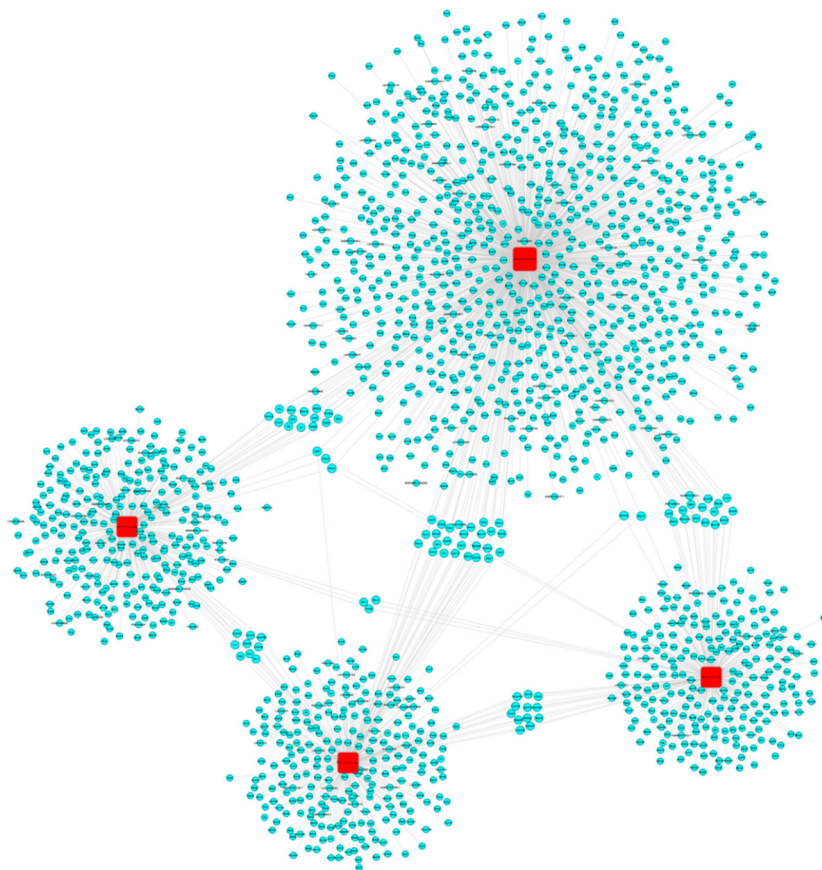

Figure S3\_up\_tRFsTarget

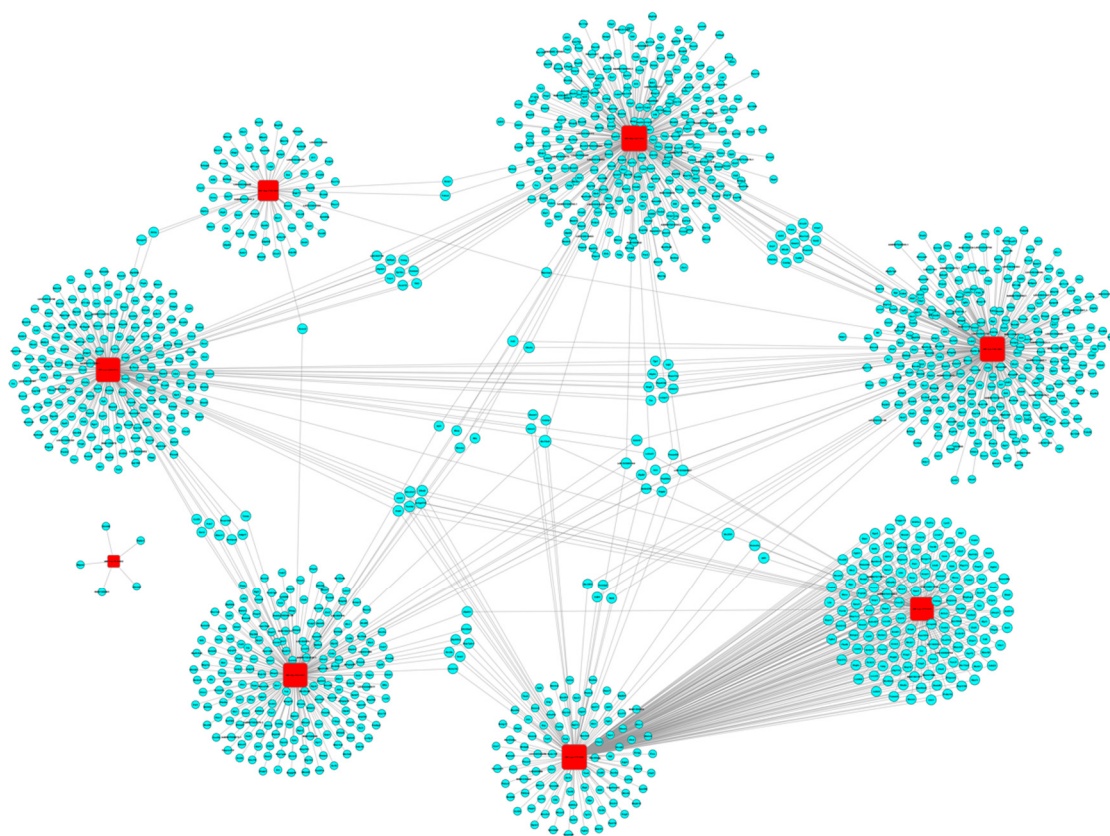

Figure S4\_down\_tRFsTarget

Supplement: Supplementary file 1 [file ijms-24-10323-s001.zip › ijms-2404127-supplementary.pdf]
